# Modelling segmental duplications in the human genome

**DOI:** 10.1186/s12864-021-07789-7

**Published:** 2021-07-02

**Authors:** Eldar T. Abdullaev, Iren R. Umarova, Peter F. Arndt

**Affiliations:** 1 Department of Computational Molecular Biology, Max Planck Institute for Molecular Genetics, Ihnestraße 63/73, Berlin, 14195 Germany; 2 Faculty of Computational Mathematics and Cybernetics, Moscow State University, Leninskiye Gory 1-52, Moscow, 119991 Russia

**Keywords:** Segmental duplications, SDs, Complex networks

## Abstract

**Background:**

Segmental duplications (SDs) are long DNA sequences that are repeated in a genome and have high sequence identity. In contrast to repetitive elements they are often unique and only sometimes have multiple copies in a genome. There are several well-studied mechanisms responsible for segmental duplications: non-allelic homologous recombination, non-homologous end joining and replication slippage. Such duplications play an important role in evolution, however, we do not have a full understanding of the dynamic properties of the duplication process.

**Results:**

We study segmental duplications through a graph representation where nodes represent genomic regions and edges represent duplications between them. The resulting network (the SD network) is quite complex and has distinct features which allow us to make inference on the evolution of segmantal duplications. We come up with the network growth model that explains features of the SD network thus giving us insights on dynamics of segmental duplications in the human genome. Based on our analysis of genomes of other species the network growth model seems to be applicable for multiple mammalian genomes.

**Conclusions:**

Our analysis suggests that duplication rates of genomic loci grow linearly with the number of copies of a duplicated region. Several scenarios explaining such a preferential duplication rates were suggested.

**Supplementary Information:**

The online version contains supplementary material available at (10.1186/s12864-021-07789-7).

## Background

Segmental duplications (SDs) are conventionally defined as long duplications of a genomic region (>1 kbp) within one genome and with a relatively high level of sequence identity (>90*%*). Segmental duplications should not be confused with simple genomic repeats which are present in higher copy number, are usually shorter, and have other mechanisms of propagation in a genome (Sup. Figure 1). In the human genome, the segmental duplications defined this way mostly comprise recent events that happened after the divergence of the New and Old World monkeys [1, 2]. The copied segments might be located on the same chromosome (intrachromosomal) or different chromosome (interchromosomal). Seminal studies found that about 5% of the human genome is actually part of segmental duplications, however this fraction can be even higher according to recent estimates [3–5]. Segmental duplications play an important evolutionary role by being involved in gene duplication and changes in regulatory sequences of genes. There are several human-specific duplicated genes, such as: *ARHGAP11B* and *SRGAP2C* that were involved in human brain evolution [6, 7]. Moreover, it was shown that genomic loci that are duplicated most frequently in SDs (core duplicons) are enriched with genes rapidly evolving in the human and great ape lineages [8–12]. Further, SDs are often involved in disease-causing rearrangements and copy-number variations in human population [1, 13].

The task of annotating SDs in a genome is not straightforward. There are two widely accepted methods for SDs detection that were first used on the draft human genome by Eichler’s group: whole-genome assembly comparison (WGAC) and whole-genome shotgun sequence detection (WSSD). In a nutshell, WGAC is a BLAST-based method that identifies paralogous sequences in a reference genome [3]. However, misassembly errors which might be attributed to SDs have to be taken into account, and therefore an experimental validation is needed for proper SD annotation. This problem is especially prominent for non-human reference genomes because their assemblies are often of a lower quality [14]. WSSD is another method that identifies genomic regions of high read coverage and relatively low mapping quality by aligning whole genome shotgun sequencing reads to the reference genome [4].

Segmental duplications are not uniformly distributed in the genome; some regions are duplicated many times while other are depleted of duplications. For example, the genomic regions proximal to centromers and telomeres are enriched with segmental duplications. Moreover, it was observed that localization of segmental duplications can be associated with other genomic features, such as: simple genomic repeats (especially *Alu* ones), increased GC content sequences, regions of lower recombination rates, fragile DNA sites etc. [15–17]. Also segmental duplications are often enriched with copy-number variations. This likely happens because of genomic instability or reduced purifying selection acting on CNVs in those regions [18, 19].

There are three main mechanisms responsible for segmental duplications: non-allelic homologous recombination (NAHR), replication slippage (or template switching) and non-homologous end joining (NHEJ). The mechanisms of segmental duplications vary in different parts of chromosomes. Subtelomeric SDs are enriched with interchromosomal duplications and are mostly produced by NHEJ [20]. Similarly, pericentromeric SDs are enriched by interchromosomal duplications, but at least 30% of all pericentromeric duplicated sequence can be traced to ancestral duplication segments (duplicons) originating from other parts of chromosomes [21]. The two-step model was proposed to explain such a mosaic structure of pericentromeric SDs. Firstly, the donor loci interspersed throughout the genome are transposed into one acceptor locus while in the second step, the acceptor locus is copied partially or completely in mosaic blocks [22, 23]. The remaining SDs are called interstitial SDs and are enriched with intrachromosomal and tandem duplications. *Alu* repeats are often observed in flanking regions of those SDs. It was suggested that propagation of segmental duplications in the ancestral human genome was associated with the burst of *Alu* retroposition about 35–40 million years ago [1, 15]. Later those SDs themselves became the source of homology for further duplication events [24, 25]. Non-allelic homologous recombination was suggested as the major mechanism for interstitial duplication events, however, later it was observed that replication based mechanisms also play an important role in SDs formation [26, 27]. Accurate prediction of a mechanism responsible for duplication events in a specific locus is often a complicated task which involves accurate inspection of local sequence features, such as: microinsertions/deletions, search for stretches of homology and ancestral regions.

Even though a lot of attention has been paid to various scenarios of SDs formation and reconstruction of complex duplication events in mosaic loci, not that many attempts were made to study global dynamical aspects of SDs propagation in the genome. There were several attempts to study the past evolution of SDs in the human genome using some mathematical approaches [5, 8, 17]. In one prominent example A-Bruijn graphs were used to study segmental duplications as a set of duplication blocks divided by breakpoints. Most actively duplicated blocks (core duplicons) were predicted and studied in a context of human evolution [8].

Our goal is to find a mathematical model for segmental duplications propagation in the human genome to explain the dynamical properties of this process. We based our analysis on a network representation of SDs, which allows us to make use of tools and concepts in network analysis. For example we can study distributions of different network characteristics, such as: node degrees, i.e. number of edges that a node has or sizes of connected components, i.e. isolated subgraphs where path between any pair of nodes exists. We can simulate synthetic network growth according to some predefined rules to suggest the model of how the network of segmental duplications evolved. Our aim is to find a “minimal model” which can reproduce key network features of the observed SD network with only a few simple rules of network growth. Through this analysis we infer certain dynamical aspects of segmental duplications propagation in a genome.

We start our analysis with a very simple model which includes only a random copy-paste process with constant duplication rate. As it turns out such a simple model cannot explain certain features of the SD network and it seems like duplication rates of genomic regions grow with the number of copies of that region and some biological explanation for this observation was suggested.

## Results

### Network construction

We based our analysis on segmental duplications (SDs) in the human genome, which have been previously identified and can be downloaded from the UCSC genome browser [3]. Basically, we start from a list of pairwise local alignments longer than 1 kbp with at least 90% identity between different regions of the human reference genome. There are 27348 autosomal alignments in this list. However, not every reported alignment refers to a unique segmental duplication event, because, when a new duplication overlaps with an older one, the new copy aligns not only to the ancestral region, but also can be aligned to other copies of the ancestral region. In general, we call such an alignment “secondary” when it appears as a result of an overlap between a new duplication and an already duplicated region.

**Fig. 1:**
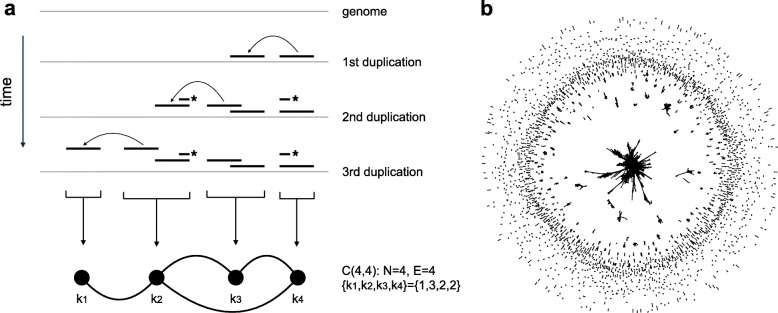
**a**. The scheme illustrates an example of several duplication events in the genome, the resulting alignments and the network constructed based on those alignments. In every time step one duplication happens in the genome and a second copy is inserted in the genome nearby. Alignments appear not only between a copied region and its copy as expected, but also when a duplication overlaps one of existing duplicated regions (the second duplication event on the scheme). We call those alignments “secondary” alignments because they do not represent a duplication event between aligning regions. For the network construction we grouped sets of overlapping alignments into separate duplicated regions. Each duplicated region is represented with a node in the SD network. Edges are added if there exists an alignment between duplicated regions. Number of edges that a node has (node degree) represents a number of copies of a corresponding duplicated region. **b**. The network constructed based on SDs of the reference Human genome (SD network). The black circles and lines represent nodes and edges of the SD network. There are 6656 nodes and 16042 edges in the SD network in total. One can see that the SD network includes multiple small connected components and a distinctive giant component with 1325 nodes and 9678 edges in it

To study this puzzling system of segmental duplications (Sup. Figure 1) we generated a network of SDs in the following way: each node represents a genomic region that is covered by (a maximal set of) overlapping alignments. Undirected edges link nodes if an alignment between two regions exists (Fig. 1a). In the remainder of the text we will denote genomic regions that correspond to nodes of the SD network as duplicated regions and will associate network characteristics to those regions directly, for example, we consider a number of neighbors of a node as a node degree of the corresponding duplicated region.

### Network characteristics

The resulting SD network has 6656 nodes and 16042 edges (Fig. 1b). The network can be decomposed into 1999 connected components, i.e. isolated subgraphs where any pair of nodes is connected by a path of edges. One distinctive feature of the SD network is that it includes a giant component with 1325 nodes (19.9*%* of all nodes) and 9678 edges (60.3*%* of all edges) that corresponds to multiple overlapping duplication events enriched in some genomic loci.

**Fig. 2:**
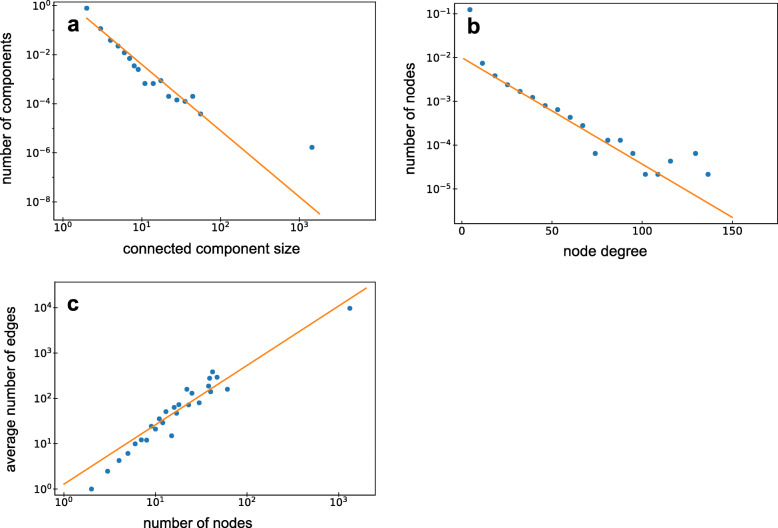
**a**. The SD network component size distribution plotted on a log-log scale using logarithmic binning (see “Methods” section). The number of connected components decreases with their size comparable to a power-law distribution *p*(*N*)∝*N*^−2.7^ which is represented as a straight orange line added as a guide to the eye. One distinctive feature of this distribution is the presence of a giant component which shows up as a single dot on the right of the distribution. **b**. The node degree distribution of the SD network plotted on a log-linear scale. An exponential tail of the node degree distribution is stressed with the orange guide to the eye line. **c**. For each component size observed in the SD network the average number of edges in corresponding components is plotted on a log-log scale. An average number of edges in components grows as a power-law of a component size: *E*(*N*)∝*N*^1.47^ dependence (orange line) was fitted with linear regression log(*E*)∼ log(*N*)

This network can be further described considering topological network characteristics, for example the component size distribution (Fig. 2a), which decreases following a power-law distribution *p*(*N*)∝*N*^−2.7^ while the giant component is well separated from this distribution. The distribution of node degrees, i.e. the number of edges a node has, has a mean of 4.8 and has an exponential tail for large node degrees (Fig. 2b). Interestingly, the average number of edges *E* in a component with *N* nodes follows a power-law: *E*(*N*)∝*N*^1.47^ (Fig. 2c). Later we will come back to this observation and give an interpretation of it.

Due to its size we can study the giant component in more detail. The clustering coefficient of a node is the number of edges between vertices in the neighborhood of the node divided by the overall number of possible edges between those vertices. The mean clustering coefficient calculated over all nodes in the giant component was equal to $\overline {C} = 0.57$ in the SD network. The average shortest path length *l*=4.93. The modular structure of the giant component was also investigated using the label propagation algorithm [28]. It was found that the giant component is enriched with “dense” clusters of nodes or “modules”. A close inspection of these modules shows that the majority of network modules were enriched with intrachromosomal duplications (most of nodes in a module belong to the same chromosome).

Even though the SD network can be described by general topological features we want to remark that the observed topology does not coincide with one of the well-studied network topologies (like scale-free or random networks) [29]. We therefore decided to simulate the dynamics of a network growth based on some predefined “rules” inspired by our knowledge on genome evolution to see if such a synthetically generated network might reflect the same network topology as the observed SD network.

### Dynamical processes

In order to study dynamical aspects of the propagation of SDs in the human genome we first constructed SD network and then asked what dynamical process could generate such a network. We decided to simulate possible network growth models that were inspired by copying models [30]. Finding a simple network growth model that would generate similar features and topology as the SD network would shed light on the dynamical processes of how the SD network evolved.

**Fig. 3:**
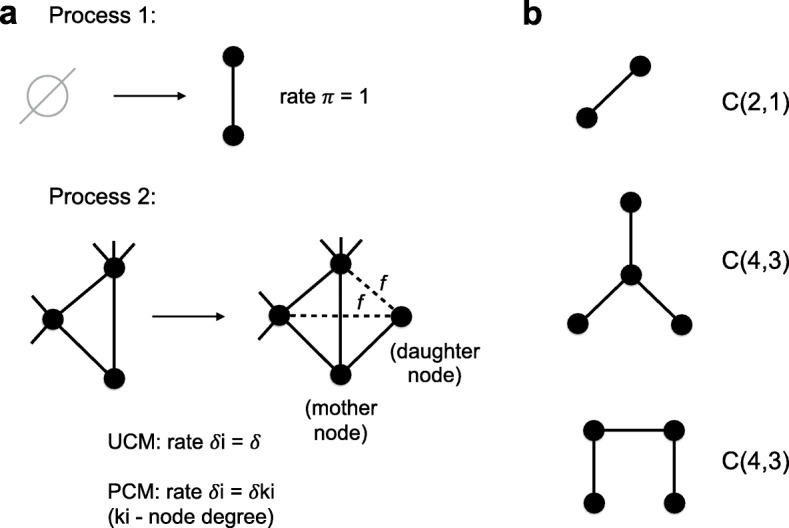
**a**. The scheme illustrates two processes of a network growth in our growth models. One can find a biological explanation for these two processes in the main text. Process 1: The component *C*(2,1) is added to a network with the rate *π*. Process 2: Each node *i* in the network can be duplicated with the rate *δ*_*i*_. We call the pre-existing to be duplicated node a “mother” node, while the new node is called a “daughter” node. A “daughter” node gets at least one edge linked to a “mother” node by default and inherits connections from the “mother” node to its neighbors each with the probability *f*. In other words, each neighbor of a “mother” node can become a neighbor of a “daughter” node with the probability *f*, while the edge between “mother” and “daughter” nodes is always added. The difference between the Uniform Copying Model (UCM) and the Preferential Copying Model (PCM) is in defining the duplication rates of nodes *δ*_*i*_. In the UCM the node duplication rates are constant: *δ*_*i*_=*δ* for all nodes, while in the PCM the duplication rates are linearly proportional to a node degree of the corresponding node: *δ*_*i*_=*δk*_*i*_ where *k*_*i*_ is a node degree of the *i*th node. **b**. We denote components with *N* nodes and *E* edges as *C*(*N*,*E*). This notation does not always correspond to a unique possible graph topology, for example, there is only one topology for *C*(2,1) while there are two for *C*(4,3). Components with *N* nodes and any possible number of edges are denoted as *C*(*N*,∗) which is the same as all components of size *N*
- The second process represents duplication events that overlap existing duplicated regions and thus new copies acquire not only alignments with the ancestral duplicated regions, but can also give rise to secondary alignments with the other copies of the duplicated region (Fig. 1a). If the overlap is long enough we expect it to be annotated as a segmental duplication even though it corresponds to a secondary alignment. In the context of network growth this process is represented by a duplication of an existing “mother” node (that by definition has copies elsewhere in the genome) and the new “daughter” node inheriting some fraction of neighbors from the “mother” node in addition to the edge between the “mother” and the “daughter” nodes that is added by default (Fig. 3). In our probabilistic model we added a parameter *f* that represents the probability of each edge connected to the “mother” node to be inherited by the “daughter” node. After a duplication the node degree of a “daughter” node $k_{d} \in 1,2,\dots,k_{m} + 1$ where *k*_*m*_ is the node degree of a “mother” node (Fig. 3).
  Node duplications happen with the rates proportional to a second parameter *δ*. However, since only the ratio of the two rate parameters *δ*/*π* matters for simulations we assume *π*=1 in the remainder of the text (see “Methods” section).

Our network growth model includes two processes:

- The first process represents novel duplications that do not overlap any older ones. In the context of network growth this results to the de novo addition of a connected components *C*(2,1) (i.e. with 2 nodes and 1 edge) with rate *π* to the network (Fig. 3).

#### The uniform copying model (UCM)

**Fig. 4:**
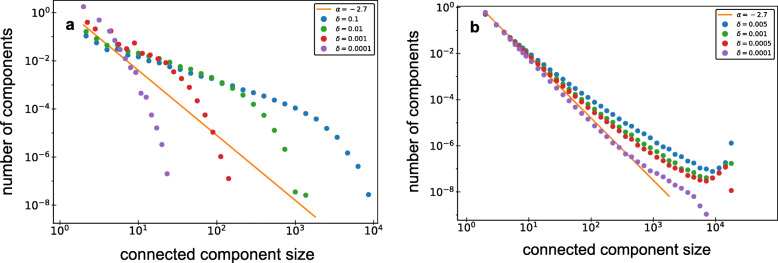
The connected component size distributions observed in pooled (see “Methods” section) simulations of network growth based on UCM and PCM growth models. In all cases we used the parameter *f*=0.5 and *δ* values listed in the legend. An orange guide to the eye line is added to illustrate the slope observed in the connected component size distribution of the SD network (*p*(*N*)∝*N*^−2.7^). **a**. The component size distributions observed in the UCM simulations differ from the one observed in the SD network. Both slopes are different and no peaks that correspond to giant components are observed in the UCM simulated networks. **b**. The component size distributions observed in the PCM simulated networks are similar to the one of the SD network. All distributions independently of *δ* value follow a similar slope on a log-log scale to the one of the SD network for component sizes observed in the SD network. Moreover, both the PCM synthetic networks and the SD network include giant components. It can be seen as a peak at the right side of the component size distributions of the PCM simulated networks

The node duplication rate can be parameterized in multiple ways. In the simplest model, we assume that the duplication rate for all nodes is the same, *δ*_*i*_=*δ*. We will further refer to this model as the Uniform Copying Model or UCM (Fig. 3). The connected component size distributions in networks grown using the UCM follows a power-law distribution *p*(*N*)∝*N*^−1^. Although disguised by finite-size effect in Fig. 4a, this can be more clearly seen in longer simulations in Sup. Figure 2. In the Supplementary text we also derive this behavior analytically. Since the connected component size distributions of synthetic networks are different from the one of the SD network (the power-law exponents are different and they lack prominent giant components) we assume that the SD network evolved according to another network growth model.

#### The preferential copying model (PCM)

The UCM was not sufficient to explain the SD network topology. This motivated us to study a different dynamics of copying models. The next simplest copying model is the one where a duplication rate of a node *i* depends linearly on the node degree *k*_*i*_: *δ*_*i*_=*δk*_*i*_. In this copying model highly connected nodes will be duplicated with preference and we will further refer this model as the Preferential Copying Model or PCM (Fig. 3).

Our analytical solution predicts the power-law distribution *p*(*N*)∝*N*^−1−*f*^ for the connected component size distribution. This behaviour is also observable in simulations of the PCM. The power-law tail gets obvious for pooled long simulations (see Sup. text, Sup. Figure 2).

There is no reason to reject the PCM based on the connected component size distributions observed in synthetic networks. In all the PCM simulations observed component size distributions followed a similar slope on a log-log scale to the one of the SD network (Fig. 4b). Moreover, giant components were present in the PCM simulated networks similarly to the SD network (Fig. 4b).

### Estimation of the parameters for the PCM

To make further conclusions on relatedness of the PCM to the evolution of the SD network, we inferred values for the parameters *f* and *δ* such that a PCM generated network matches the characteristics of the observed SD network.

The average fraction of neighbors *f* inherited from a “mother” node was predicted using an interesting observation. We observed that the average number of edges *E* in connected components generated by the PCM grows with the number of nodes *N* according to *E*∝*N*^1+*f*^ when *N*→*∞*. This is in contrast to a more complicated dependence that can be analytically predicted for simpler UCM (see Sup. text, Sup. Figure 3). We therefore used a linear regression of log(*E*)∼ log(*N*) to estimate the power-law exponent and find that the power-law *E*∝*N*^1.47^ fits best to the observations, thus suggesting the value *f*_reg_=0.47 (Fig. 2c). The parameter *δ* was predicted using Approximate Bayesian Computation (ABC) to be equal to *δ*_ABC_=5.1∗10^−4^ with the 95% confidence interval for the parameter value: *δ*_ABC_∈[3∗10^−4^;6.6∗10^−4^] (see “Methods” section).

Independent of the above methods, an alternative method was applied to infer the values of *f* and *δ* parameters. Based on the PCM we expect that when a duplication happens in a component *C*(2,1) we get either a component *C*(3,3) or *C*(3,2) with probabilities *f* and 1−*f*, respectively. Moreover, according to the PCM an overall rate of further duplications in *C*(3,3) is 1.5 times higher than in *C*(3,2) components because the sum of node degrees equals 6 and 4, respectively. All bigger components *C*(> 3,∗) appear as a result of one or more duplications in *C*(3,∗) components. New *C*(2,1) components appear with the rate *π*=1. For a mathematical analysis of the temporal dynamics we will denote the expected numbers of such components at time *t* as *n*_*t*_(2,1),*n*_*t*_(3,2),*n*_*t*_(3,3) and *n*_*t*_(> 3,∗) respectively. As described above their time dependence is given by the following set of partial differential equations:

$$\begin{array}{@{}rcl@{}} \frac{\partial n_{t}(2,1)}{\partial t} &=& 1 - 2 \delta n_{t}(2,1), \\ \frac{\partial n_{t}(3,2)}{\partial t} &=& 2 \delta (1-f) n_{t}(2,1) - 4 \delta n_{t}(3,2),  \\ \frac{\partial n_{t}(3,3)}{\partial t} &=& 2 \delta f n_{t}(2,1) - 6 \delta n_{t}(3,3),  \\ \frac{\partial n_{t}(>\!3,*)}{\partial t} &=& 4 \delta n_{t}(3,2) + 6 \delta n_{t}(3,3)  \end{array} $$

This system of equations leads to:

$$\begin{array}{@{}rcl@{}} n_{t}(2,1) &=& \frac{\left(1 - e^{-2 \delta t}\right)}{2\delta}, \\ n_{t}(3,2) &=& \frac{(1 - f)\left(1 - 2e^{-2\delta t} + e^{-4\delta t}\right)}{4\delta},  \\ n_{t}(3,3) &=& \frac{f\left(1 - 1.5e^{-2\delta t} + 0.5e^{-6\delta t}\right)}{6\delta},  \\ n_{t}(>\!3,*) &=& \frac{f \!- 9 + 3(4\!-f)e^{-2\delta t} \!- 3(1\!-f)e^{-4\delta t} \!- fe^{-6\delta t}}{12\delta}  \end{array} $$

There are 4 equations and 3 unknown variables *f*,*δ* and *t* in this system. Therefore the goal is to find *f*,*δ*,*t* values that minimize a certain loss function. Here we used the weighted city block distance *L*:

$$L = \sum_{i=1}^{4} \frac{| \vec{n}_{t,i} - \vec{n}_{\text{sd}, i} |}{\vec{n}_{\text{sd},i}} $$

between the following vectors:

$$\vec{n}_{t} = \left(\begin{array}{l} n_{t}(2,1) \\ n_{t}(3,2) \\ n_{t}(3,3) \\ n_{t}(>\!3,*) \end{array}\right) \text{ \quad and \quad } \vec{n}_{\text{sd}} = \left(\begin{array}{l} n_{\text{sd}}(2,1) \\ n_{\text{sd}}(3,2) \\ n_{\text{sd}}(3,3) \\ n_{\text{sd}}(>\!3,*) \end{array}\right) $$

where *n*_sd_(*N*,*E*) is a number of components *C*(*N*,*E*) in the SD network. Minimization of a loss function *L* over *f*,*δ* and *t* variables was performed with the Nelder–Mead method which converged to its minimum at *f*_min_=0.52; *δ*_min_=3.2∗10^−4^; *t*_min_=1320.

Both independent methods: regression/ABC and minimization result in consistent predictors of the model parameters (*f*_reg_=0.47, *δ*_ABC_=5.1∗10^−4^) and (*f*_min_=0.52, *δ*_min_=3.2∗10^−4^). However, for future simulations we will use only the *f*=*f*_reg_=0.47 and *δ*=*δ*_ABC_=5.1∗10^−4^ values.

**Fig. 5:**
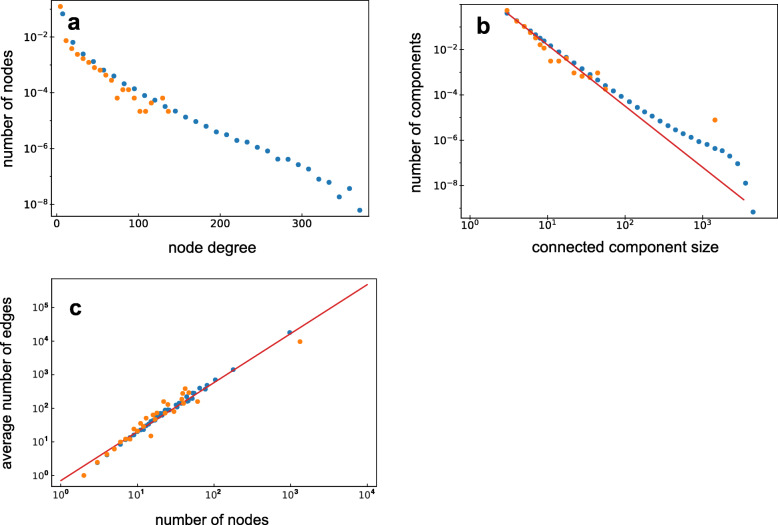
Different topological characteristics of the SD network (orange dots) and the PCM simulated networks with inferred parameters *f*=0.47,*δ*=5.1∗10^−4^ (blue dots) are compared. Multiple PCM simulations were pooled together to get a better resolution for the distributions. **a**. The node degree distribution is plotted on a log-linear scale with the linear binning. We can see that the exponential tail is observed in both synthetic and the SD networks and the power of exponents is the same. **b**. The connected component size distributions plotted on a log-log scale with the logarithmic binning. The slope observed in simulations is the same as the one of the SD network for all component sizes observed in it. The peak that corresponds to the giant component is also present where expected. **c**. The average number of edges in components of different sizes is plotted against a component size on a log-log scale. We can see that the average number of edges grows as a power-law of a component size: *E*∝*N*^1.47^ (red line) in simulated and the SD networks

Different topological characteristics of the PCM simulated network (*f*=0.47;*δ*=5.1∗10^−4^) were compared with ones of the SD network (Fig. 5). Those networks are very similar in both connected component size and node degree distributions. Moreover, we have no reason to reject the hypothesis that the giant component of the SD network comes from the distribution of the biggest components of the PCM synthetic networks (empirical *p*-value = 0.21) (Sup. Figure 4).

Moreover, characteristics of the giant component in the SD network were compared with other previously studied randomly generated networks, i.e. the configuration model network (random network of a given degree sequence), random graph, scale-free network and the giant component of the PCM synthetic network. All these networks were of the same or comparable size (number of nodes and edges) as the SD network (see “Methods” section). The giant component of the SD network is more similar to the giant components observed in the PCM simulations than to the other networks that we used in comparison (Table 1).

**Table 1:** Different characteristics of Erdős–Rényi random graph, scale-free network, configuration model network (the same node degrees as in the giant component of the SD network) and the giant components (GC) observed in the SD and PCM simulated networks are compared

Type	Clustering coefficient	Shortest path
SD network GC	0.57	4.93
PCM network GC	0.18	3.5
Random network	0.012	2.95
Scale-free network	0.031	2.83
Configuration network	0.08	3.02

These characteristics include: a mean clustering coefficient and an average shortest path length. All networks/components in our comparison were of the same size (see “Methods” section). Among the networks we studied the PCM synthetic network is the most similar to the SD network (even though these are rather distinct)

Overall, we found convincing evidence that the PCM growth results in networks topologically similar to the SD network. This means that the probability of a duplicated region to be duplicated again grows linearly with the number of copies (node degree) of that region. More precisely, the duplication probability grows linearly with the number of loci that share long homologous sequences with the region, including secondary alignments. There are several possible explanations for such growth.

### Reasons for the preferential copying model

The length of duplicated regions could be a major factor explaining why duplication rates grow linearly with node degree. We may expect that the probability of a duplicated region to overlap a new SD would grow with the length of the duplicated region. We therefore studied the effect of length on the duplication rates and the factors influencing it. One of the ways to find such factors is to select those variables (features of duplicated regions) that are important in the prediction of a response variable, i.e. the length of duplicated regions. By using the random forest regression and permutation tests we found that there are several characteristics of duplicated regions that significantly (*p*-value <0.01) affect its length, such as: number of double edges and self-loops in the unfiltered SD network, node degree and mean copy-number of a duplicated region (see “Methods” section for details).

For these significant factors we can reason why they play a role in our problem. With every new duplication of a duplicated region (which effects its node degree and mean copy-number) or duplication that “jumps” into an already duplicated region (effects the number of self-loops and double edges) we expect an increase of a duplicated region length. So the length of a duplicated region is influenced by the interplay of several factors, however, we can see that the mean length of a duplicated region grows linearly with the node degree of a duplicated region (Sup. Figure 5). Thus we can assume a mechanistic explanation: the preferential duplication rates appear because the probability of a new SD to overlap a duplicated region is higher for longer duplicated regions.

The node degree represents the number of long sequences in other genomic loci homologous to a corresponding duplicated region. These stretches of long homology increase the probability for genomic rearrangements (including duplications). Thus with growing node degree the probability of a duplicated region to be involved in homology-mediated genomic rearrangements also grows and that might be another factor explaining the preferential duplication rates of the PCM.

In the previous sections we studied only the SDs that were fixed in the human genome. However, the fixation process of new duplications can also be affected by the SDs that were duplicated before. To study this effect copy-number variations (CNVs) observed in 2504 individuals were downloaded from the 1000 Genomes project [31]. All autosomal CNVs were split into 3 groups based on their frequency in the human population. There were rare, medium and high frequency CNVs with corresponding minor allele counts (MACs) in three ranges: [1; 3], [4; 15] and [16; 2504]. The duplicated regions (nodes) were also split into 4 groups according to their node degree in the SD network: [1; 1], [2; 5], [6; 30] and [31; 140]. In both cases the intervals were chosen such that the number of observations in each interval is comparable. Since both distributions are highly skewed towards small values the intervals get longer for larger values.

**Fig. 6:**
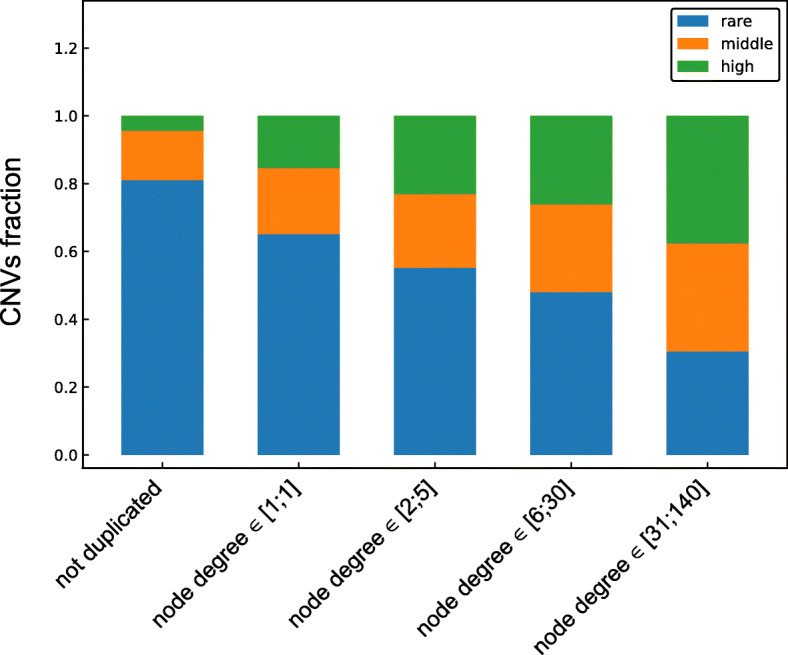
The characteristics of CNVs that overlap different genomic regions. These genomic regions include duplicated sequences of different node degrees (specified on x axis) and the remaining not duplicated parts of the genome. CNVs that overlap different genomic regions are split into 3 groups based on a frequency and their fractions are plotted in different colours. Rare CNVs (1≤ MAC ≤3), medium frequency CNVs (4≤ MAC ≤15) and high frequency CNVs (16≤ MAC ≤2504) are colored in blue, orange and green respectively. The fraction of high frequency CNVs is higher in all duplicated regions than in the rest of the genome and this fraction grows with the node degree of duplicated regions

For duplicated regions that belong to each group we studied frequencies of all CNVs that overlap those regions (Fig. 6). We can see that medium and high frequency CNVs are enriched in duplicated regions in comparison with the rest of the genome. Moreover, the fraction of high frequency CNVs grows with a node degree of a duplicated region, while the fraction of rare CNVs decreases. This can result from interactions with multiple factors, such as: the local duplication rates, recombination rates, reduced purifying selection in highly duplicated regions etc. (see “Discussion” section). However, it is likely that the probability of a CNV to be fixed in a population is higher if it overlaps high node degree duplicated region. This might be another factor explaining the preferential duplication rates of the PCM.

### SD networks of other species

With more than 90% identity between copies the segmental duplications we detect today in our genome appeared about 40 million years ago or later if we assume neural evolution, roughly corresponding to the timeline after the divergence of the New and Old World monkeys [1]. We therefore got interested in the question whether the SD networks of other species which evolved independently from humans are similar to the human SD network or may have resulted from other growth scenarios. We therefore downloaded the latest reference genomes of 8 additional species: gorilla (*Gorilla gorilla gorilla*), gibbon (*Nomascus leucogenys*), mouse (*Mus musculus*), rat (*Rattus norvegicus*), dog (*Canis lupus familiaris*), chicken (*Gallus gallus*), zebrafish (*Danio rerio*) and worm (*Caenorhabditis elegans*). The SEDEF tool was used to de novo annotate SDs in the genomes of these species (see “Methods” section) [32]. For comparison we also used the same tool for the human genome. Based on the SDs identified by SEDEF the SD networks were constructed for the above species including human. The human SD network built from SEDEF predicted SDs was compared with the original one (Sup. Figure 6a). The SEDEF predicted SD network is larger both in terms of the number of nodes and edges (Table 2), however, almost all duplicated regions from the original SD network are present in it (see “Methods” section). Moreover, the original and SEDEF predicted human SD networks are similar in all topological characteristics that we studied including the slope of the connected component size distribution (Sup. Figure 6a).

**Table 2:** Characteristics of the SD networks of different species

	Human	Gorilla	Gibbon	Mouse	Rat	Dog	Chicken	Zebrafish	Worm
GS (10^9^ bps)	2.88	2.78	2.65	2.46	2.62	2.2	0.96	1.35	0.083
Num. of nodes	12,579	30,935	29,376	14,766	35,919	18,438	3,169	34,445	1,572
Num. of edges	37,319	42,643	443,916	166,145	183,618	62,308	17,102	601,289	2,199
Intra- (%)	19	46	7	8	24	7	36	9	37
Tandem (%)	6	25	2	2	10	3	8	1	17
*f* value	0.48	0.43	0.57	0.42	0.42	0.33	0.29	0.35	0.32

These include: genome size (GS) excluding sex chromosomes, number of nodes, number of edges, fraction of intrachromosomal edges and tandem edges among all edges of a network and regression-based predicted *f* values. An edge is denoted as tandem if both duplicated regions linked with the edge are located at the same chromosome at the distance <5∗10^5^ bps

**Fig. 7:**
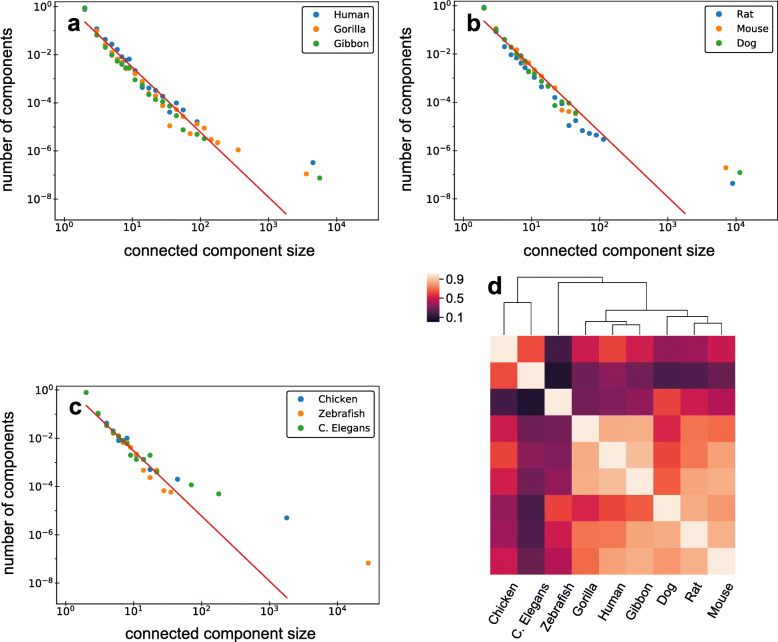
The connected component size distributions plotted for the SD networks of different species on a log-log scale. The red lines in panels (a)-(c) represent the slope observed in the SD network of human. Observed distributions follow this slope on a log-log scale and a giant component is observed in most species. **a**. The group of primate species that includes human, gorilla and gibbon. **b**. The group of other mammalian species that includes rat, mouse and dog. **c**. The group of distinct species that do not belong to mammals: chicken, zebrafish and C. elegans. The SD network of C. elegans is the smallest one, thus we do not see a prominent giant component as in other species. **d**. The heatmap of similarities (1− Bray-Curtis dissimilarities) between connected component size vectors in all studied species. The dendrogram on top corresponds to hierarchical clustering of the species according to their similarities. We can see that the dendrogram, to some extent, reflects phylogenetic relationships between species (for example, presence of primate and mammalian branches)

The resulting SD networks of different species are quite distinct in their sizes and other network characteristics (Table 2). The component size distributions, on the other hand, are similar both in terms of the slope of the distributions and in the presence of a giant component (Fig. 7). Similarly to the human SD network we also observed a power-law growth of the average number of edges with component size in all species. Corresponding regression-based predicted values of the parameter *f* are listed in the Table 2. When we calculated Bray-Curtis pairwise dissimilarities between connected component size vectors we found that the species cluster similarly to their phylogenetic relationships, for example, primate and mammalian clusters are present (see “Methods” section) [33]. We note that the shared common SDs cannot be responsible for such similarities between the SD networks of relatively distant species. Thus, based on our data, the topology of an SD network seems to be reflective of phylogenetic relationships among species (Fig. 7d) indicative of shared slowly evolving molecular mechanism responsible for the continuous spread of segmental duplications.

## Discussion

We made an effort to study the dynamics of a globally acting propagation process for segmental duplications in the human genome. To do this we came up with a mathematical formalization in terms of networks and network growth processes. We generated the SD network from annotated SDs; in this network nodes represent genomic regions and edges indicate the presence of a segmental duplication. This gave us the opportunity to investigate several network growth models and reason about their relevance in describing the naturally observed process. The simplest copying model with equal probabilities of node duplications (UCM) is not sufficient to explain the SD network topology. However, a more complicated preferentially copying model (PCM) with preferential node duplication rates nicely fits all topological characteristics of the SD network, especially if taking into account that the growth model includes only 2 parameters (*f* and *δ*). Based on the PCM the duplication rate of already duplicated regions grows linearly with the number of copies of those regions (more precisely, with the number of loci that share long homologous sequences with those regions).

The PCM was accurate in predicting the SD network characteristics even without inclusion of additional processes that reflect real life events, such as: deletions of duplicated regions (the loss of nodes in the SD network), decrease of homology below the detection threshold (the loss of edges), duplication where a new copy jumps into an already duplicated region (emergence of new edges between existing nodes). Moreover, we did not include separate processes that correspond to different duplication mechanisms as they are described in the literature (duplication dynamics is different in pericentromeric, subtelomeric and other genomic regions) or different processes corresponding to intra- and inerchromosomal duplications (see “Background” section). The explicit addition of such processes to the model would make the parameterization heavier while biological conclusions more vague. However, the two-step model explaining the mosaic structure of pericentromeric SDs assumes that genomic segments are first translocated into one genomic locus (see “Background” section) and this process is not included in the PCM. We found that exclusion of pericentromeric SDs from the analysis does not change the SD network topology substantially thus keeping our predictions valid (Sup. Figure 6b). Moreover, the topology of the SD network does not change significantly if we try alternative strategies of the SD network construction or add new processes to network growth models (see “Methods” section).

We suggested some interpretations of why the network evolution of the SD network follows a model with preferential node attachment. A mechanistic explanation could be that with growing node degree the length of the corresponding duplicated region also grows, thus the probability that the next duplication will overlap that duplicated region also grows. Secondly, growing node degree of a duplicated region is also associated with growing probability of genomic rearrangements (including duplications) in that locus. Finally, we observed that the frequency of copy-number variations in the human population grows with the node degree of a duplicated region it overlaps with. This might be attributed to different scenarios: overall genomic instability of a duplicated region that has multiple copies, recurrent duplications happening in unstable genomic sites, decreased purifying selection against new duplications in those regions or positive selection for beneficial gene duplications and decreased recombination rates that reduce an efficiency of the purifying selection. In all such cases, CNVs in high node degree duplicated regions are more likely to be fixed in human population. This might explain the preferential duplication rates in PCM.

It was found that the SD network topology is quite consistent among relatively distant species, at least, in terms of component size distribution. Moreover, a topology of an SD network seems to be a biologically meaningful characteristic of species that reflects phylogenetic relationships and can further be studied.

One more observation that comes from the PCM is that the number of nodes in the network at some point starts to grow hyperbolically and nodes accumulate almost exclusively in the giant component. This means that in the PCM we do not have an equilibrium steady state. Thus the overall length of duplicated regions in the genome should also reach a hyperbolic growth at some point leading to an “SDs explosion”. If this prediction is correct it is curious how this problem is addressed in natural evolution of genomes without any notable signs of this effect in the topology of the SD network. One possible scenario is that selection might act only on high node degree duplicated regions (very right tail of the node degree distribution) by decreasing the probability for further duplications. However, a constant rate of nodes/edges loss would slow down but not prevent the hyperbolic growth. To stop this growth more complex scenarios have to be considered, for instance a time dependent rate of duplications or loss.

After all, we think that the network formalization of SDs is a good way to further study the evolution of SDs in human or other species. Moreover, the SD network might be useful in reconstructing (at first approximation) the order of duplication events in the genome. Based on the PCM the new “daughter” node inherits some fraction (*f*) of edges from its “mother” node, thus instead of a manual reconstruction of events (usually very complicated task) one could solve an algorithmic task of assigning directionality to edges based on neighbors of a node.

## Conclusions

We constructed the network of human segmental duplications and studied its characteristics. Through our mathematical considerations we were able to identify a model (the preferential copying model or PCM) that can while making minimal assumptions on the general dynamics reproduce the network characteristics of the SD network. The defining feature of this model is that the probability of a duplicated region to be duplicated again grows linearly with the number of its copies. We suggested several biological mechanism that could be responsible for such a behavior. Firstly, highly duplicating genomic regions are longer thus more likely to overlap next duplication events; secondly, there is fixation bias for CNVs in highly duplicated regions; and thirdly, duplicated regions serve as hotspots for genomic rearrangements mediated by additional duplications. Similar SD networks were stably reproduced for other species showing that preferential duplication processes might be universal in vertebrates.

It is well understood that gene duplication is a major force in evolution. The existence of a duplicated gene or genomic region allows evolutionary changes in protein function and regulatory circuitry otherwise not accessible [34]. Often this process is observed and analysed with the focus on one gene or locus. Our consideration on the implied network of segmental duplications allowed us to draw more general conclusions on dynamical properties of the duplication process.

## Methods

### Genomic data

We used already annotated segmental duplications (SDs) in the reference human genome in our analysis [3]. A corresponding list of GRCh38 annotated SDs was downloaded from the UCSC genome browser website (https://genome.ucsc.edu). This list includes information on the SDs, i.e. long matching sequence segments between different regions of the repeat masked reference human genome and some other metadata as for example the length of the sequences and their identity level. For our analysis we disregard the sex chromosomes because we expect different evolutionary forces to act on these chromosomes.

For our analysis of non-human genomes the corresponding reference genomes were downloaded from UCSC genome browsers. The list of reference genomes includes: hg38 (human), gorGor4 (gorilla), nomLeu3 (gibbon), mm10 (mouse), rn6 (rat), canFam3 (dog), galGal6 (chicken), danRer11 (zebrafish), ce11 (C. elegans). We used the SEDEF tool to predict SDs in these genomes (autosomes) and to compare annotated SDs from UCSC genome browser with predicted ones where possible [32]. The UCSC annotated SDs are almost entirely covered with SEDEF predicted ones. In fact, the number of SEDEF predicted SDs was always higher (Sup. Figure 6). This is due to the fact that SEDEF allows reporting of SDs not satisfying the criteria: >1kbp and >90*%* sequence identity and the usage of strict filtering criteria for UCSC annotated SDs (agreement of WGAC and WSSD methods predictions, FISH validation etc.) [3, 4].

The CNVs that were used to study a fixation process were downloaded from the 1000 Genomes project FTP server (http://ftp.1000genomes.ebi.ac.uk/vol1/ftp/phase3/integrated_sv_map/) [31]. We restrict ourselves to the autosomal CNVs in our analysis.

### Network analysis

As described in the “Results” section we generated a network of SDs. Each node of the SD network represents a duplicated region of the genome that covers a maximal set of overlapping alignments. Undirected edges connect nodes if an alignment between two duplicated regions exists (Fig. 1a). In general we used this network after trimming multiple edges between any pair of nodes (double edges) and self-loop edges. Only once we used characteristics of the untrimmed SD network as indicated in the text.

We convinced ourselves that qualitative and to some extend also quantitative properties of our network analysis stay invariant under slight changes of the used cut-offs or considering uncertainties in the definition of the exact borders of segmental duplication. For example, we constructed the SD network based on duplications with reduced length and sequence identity cut-offs (length >500 bps, sequence identity >70*%*) (Sup. Figure 7, Sup.Table 1) [5]. Moreover, we parameterized the process of merging SDs into duplicated regions to see if our SD network is stable under different strategies of its construction. To do this we considered padded SDs, i.e. we increased the annotated length of SDs by *P* padding bps on both sides. Negative or positive values of *P* resulted in shorter or longer SDs, respectively., While *P*=0 corresponds to our original merging process, considering padded SDs will generate slightly different networks, since SDs will overlap less or more often, respectively. We also checked if our predictions about network growth models are still valid if we add a process of edges loss to UCM and PCM simulations (Sup. Figure 8). To do this, at each time step of a network growth process we removed each edge of a synthetic network with pre-defined probability *r*. None of these factors affected our results substantially or changed our conclusions about dynamics of duplication process (Sup. Figure 7-9).

All the steps of our network analysis are performed using the LightGraphs.jl package in the Julia programming language [35, 36]. Except for simple feature extraction and modification of the SD network we used this package to calculate the mean clustering coefficient, the average shortest path length, study the modularity of the network, use configuration model, generate random networks using the Erdős–Rényi model and scale-free networks using the Barabási–Albert model [29, 37, 38].

To compare characteristics of different synthetically generated networks with the giant component of the SD network we considered networks of the same size (both the same number of nodes and edges where possible). There is no explicit way in the Barabási–Albert model to specify the number of edges in a resulting synthetic network. Thus we fitted the parameter value *k* (a number of edges that a new node forms with a preferential linkage) to get the size of a resulting network close to the size of the SD network giant component; this was achieved by choosing *k*=7 [29].

### Data visualization

When we plot distributions on a log-log scale logarithmic binning is used to reduce stochastic noise in the heavy tail of distributions. Bins are of the same size on a log-scale. For each bin the value *n*_*i*_/(*Nb*_*i*_) is calculated and plotted on log-scale, where *n*_*i*_ is a number of observations in *i*th bin, *N* is an overall number of observations, *b*_*i*_ is the *i*th bin length which grows exponentially with *i*.

To get a better statistics for distributions associated with synthetic networks we run 500 simulations with the same model, aggregated all networks and plotted distributions of resulting pooled networks.

The network visualization is done with the GraphPlot.jl package in Julia.

### Network growth models

We construct our models of network growth based on specific copying mechanism as described in the “Results” section. There are two types of processes happening during the network growth: an addition of a new connected component *C*(2,1) to a network or duplication of an existing node and inheritance of some fraction of its edges. Our assumption is that all genomic loci can be duplicated independently of other duplication events. Thus we used the Kinetic Monte Carlo (KMC) method to run a simulation of network growth [39].

For a graph with *N* nodes and *E* edges at time point *t*, a total of *N*+1 possible processes have to be considered. First the addition of a new component *C*(2,1), with the rate *π*, and the duplications of any one of the existing nodes, with rates *δ*_*i*_. The rates of all possible processes are represented as a vector $\vec {r}(t)$ of length *N*+1. For the UCM we use *δ*_*i*_=*δ* thus the rates vector:

$$\vec{r}_{\text{UCM}}(t) = \left(\begin{array}{l} \pi\\ \delta\\ \vdots \\ \delta \end{array}\right) $$

For the PCM we use *δ*_*i*_=*δk*_*i*_ where *k*_*i*_ is a node degree of node *i* thus the rates vector:

$$\vec{r}_{\text{PCM}}(t) = \left(\begin{array}{l} \pi\\ \delta k_{1}\\ \vdots \\ \delta k_{N} \end{array}\right) $$

One of *N*+1 possible processes at time point *t* is picked at random with probabilities proportional to the given rates $\vec {r}(t)$. An average waiting time before this event happens is exponentially distributed. It can be calculated as $\Delta t = - \ln (u)/\left (\sum _{i} \vec {r}_{i}(t)\right)$, where *u* is sampled randomly from the (0, 1] interval. Since only relative rates matter in the KMC we used *π*=1 in all simulations and fitted only the *δ* value.

All network growth simulations terminate when a number of nodes in a network reaches some predefined threshold (often the number of nodes in the observed SD network).

### Feature importance prediction

At some point we analyzed the factors that affect the length of duplicated regions. To do this we used the random forest regression algorithm where the length of duplicated regions (response variable) was predicted from several characteristics of duplicated regions (predictor variables) from the untrimmed SD network. These characteristics include: the node degree, the size of a connected component the node belongs to, the mean copy number of the duplicated region, a fraction of intrachromosomal edges from all edges of the node, the number of self-loop edges and the number of double edges. The last two characteristics can only be retrieved from the untrimmed SD network. The percent of variance explained using 10-fold cross-validation was *R*^2^∼67*%*.

We were interested in finding characteristics of duplicated regions that are important in its length prediction using the random forest algorithm. Permutation based importance values that are assigned to predictor variables by the random forest algorithm are usually affected by a number of categories and a scale of a variable. To overcome this problem the response variable was shuffled 1000 times while keeping predictor variables intact. Each time the random forest algorithm was trained on this data and all importance values for predictor variables were measured. Then for each predictor variable *i* an empirical *p*-value was calculated in the following way:

$$p = \frac{\sum_{j=1}^{N_{p}}I\left(\text{imp}_{j}^{p}[i] \ > \ \text{imp}^{r}[i]\right)}{N_{p}} $$

where *N*_*p*_ is the number of permutations, *I*() is an indicator function, imp^*p*^[*i*] and imp^*r*^[*i*] are the *i*th feature permutation based importance values observed with and without the response variable shuffling respectively [40]. At significance level *α*=0.01 the node degree, the mean copy number of a region, the number of double edges and self-loops are significant in a duplicated region length prediction.

### PCM parameters optimization

Approximate Bayesian Computation (ABC) is a Bayesian method to approximately predict posterior parameter distributions when an analytical formula for a likelihood function can’t be derived [41]. To apply ABC a rejection criteria (specific distance measure) and a tolerance level (distance threshold) are needed that allow to say if the resulting outcome of a simulation is similar to a real observation or not. In our case we compared the connected component size distributions in the SD and the PCM simulated networks. As a rejection criterion we used the Bray-Curtis dissimilarity [33].

The Bray-Curtis dissimilarity between a sorted arrays of *N* biggest connected component sizes is calculated in the following way:

$$D_{\text{BC}}(X, Y) = \frac{\sum_{i=1}^{N} \left | X_{i} - Y_{i} \right |}{\sum_{i=1}^{N} \left(X_{i} - Y_{i}\right)}. $$

We limited the number of components to *N*=500 because the Bray-Curtis dissimilarity can only be calculated for arrays of the same length. This can’t be guaranteed in our simulations because network growth simulations terminate when a number of nodes in simulated network reaches the threshold independently of connected components number. We used ABC method from the ApproxBayes.jl Julia package to run 5000 simulations of the PCM with *f*=0.47 and *δ* values taken uniformly from the interval [5∗10^−5^; 9∗10^−4^]. The rejection criterion is satisfied when the Bray-Curtis dissimilarity between component size vectors of simulated and the SD networks *D*_BC_(sim, SD)<0.2 (tolerance level). Based on ABC the parameter *δ*=5.1∗10^−4^ with 95% confidence interval being *δ*_0.95_=[3∗10^−4^; 6.6∗10^−4^].

To estimate the PCM parameter values the loss function was minimized with respect to *f*,*δ* and *t* parameters. The Nelder–Mead method was used from Optim.jl Julia package for this purpose [42, 43].

## Supplementary Information

**Additional file 1** Supplementary material

## Data Availability

All our scripts can be found at https://github.com/abdeldar/SD_network.git. The coordinates of GRCh38 annotated SDs were downloaded from the UCSC genome browser website (https://genome.ucsc.edu). From the same website we downloaded the reference genomes: hg38 (human), gorGor4 (gorilla), nomLeu3 (gibbon), mm10 (mouse), rn6 (rat), canFam3 (dog), galGal6 (chicken), danRer11 (zebrafish) and ce11 (C. elegans). The CNVs observed in the human population were taken from the 1000 Genomes project FTP server (http://ftp.1000genomes.ebi.ac.uk/vol1/ftp/phase3/integrated_sv_map/).

## Abbreviations

SD: Segmental duplication
NAHR: Non-allelic homologous recombination
NHEJ: Non-homologous end joining
CNV: Copy-number variation
MAC: Minor allele count
UCM: Uniform Copying Model
PCM: Preferential Copying Model
WGAC: Whole-genome assembly comparison
WSSD: Whole-genome shotgun sequencing detection
KMC: Kinetic Monte Carlo
ABC: Approximate Bayesian computation
