# Supplementary material for: Modelling segmental duplications in the human genome
Source: BMC Genomics. 2021 Jul 2;22:496. doi: 10.1186/s12864-021-07789-7 (PMC8254307; doi:10.1186/s12864-021-07789-7)
Supplement: Supplementary file 1 — Additional file 1 Supplementary material [file 12864_2021_7789_MOESM1_ESM.pdf]

Supplementary figures

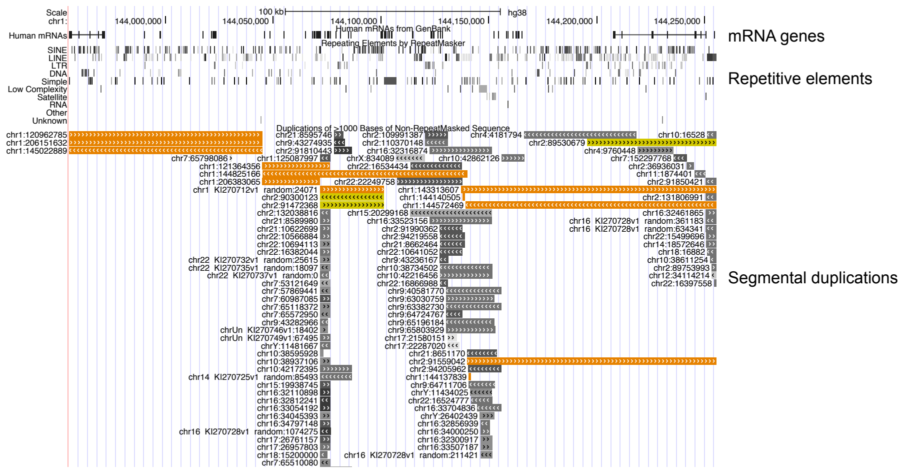

Sup. Figure 1 The UCSC genome browser annotation of an example genomic region (chr1:143,955,418-144,255,418) that allows to illustrate the difference between repetitive elements and segmental duplications. The illustrated region also shows a complex structure of genomic loci enriched with SDs. Every element of the "Segmental duplication" track represents a long alignment observed between this genomic region and the matching one located elsewhere as indicated by the coordinates specified on the left of each element. Colours of the alignments represent the level of sequence identity (light to dark grey $(90-98\%)$ - yellow $(98-99\%)$ - orange $(>99\%)$). Repetitive elements like LINE, SINE etc. are usually much shorter and present in high number of copies in the genome (see tracks on the top).

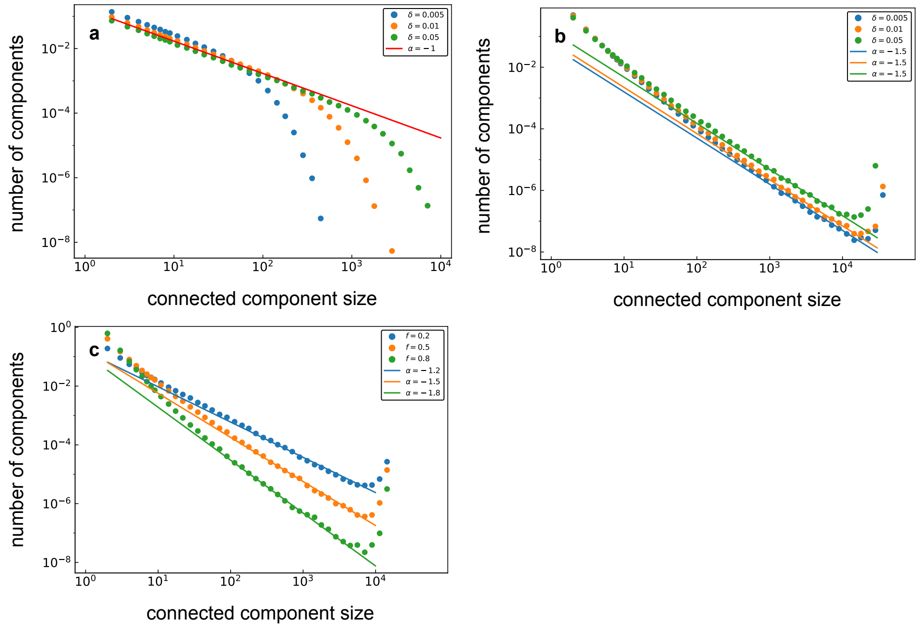

Sup. Figure 2 The connected component size distributions observed in simulations of network growth are plotted on a log-log scale. The distributions observed in synthetic networks follow our analytically predicted slopes. **a**. The UCM simulated networks follow the power-law $p(N) \propto N^{\alpha}$ where $\alpha = -1$ for all parameter δ values (parameter f values do not effect the distributions in the UCM). **b-c**. The PCM simulated networks follow the power-law $p(N) \propto N^{\alpha}$ where $\alpha = -1 - f$ for all parameter δ and f values. Straight lines represent analytically predicted slopes in all panels.

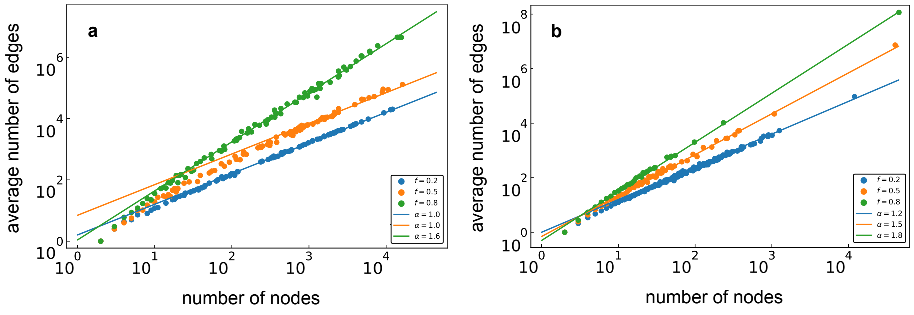

Sup. Figure 3 The average number of edges in components is plotted against a component size on a log-log scale. Different colours correspond to network growth simulations with different f values using **a** the UCM and **b** the PCM growth. Straight lines represent the slopes of a power-law growth predicted analytically for the UCM (see Analytical solutions) and observed in the PCM simulations ($E \propto N^{1+f}$).

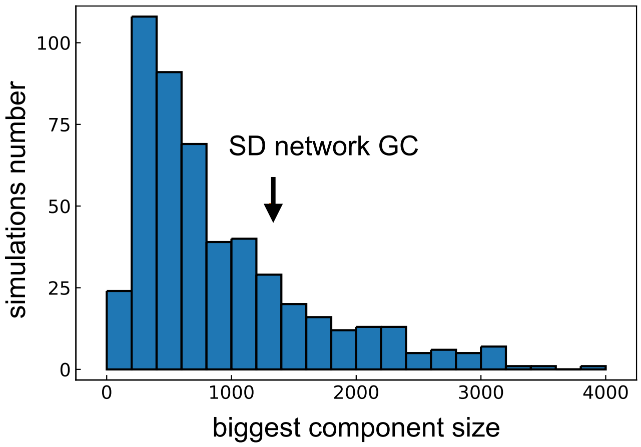

Sup. Figure 4 The histogram of sizes of the biggest components observed in the 500 PCM simulations. The arrow points to the size of the giant component of the SD network. The empirical p-value $= 0.21$, i.e. there is no reason to assume that the giant component (GC) of the SD network comes from another distribution.

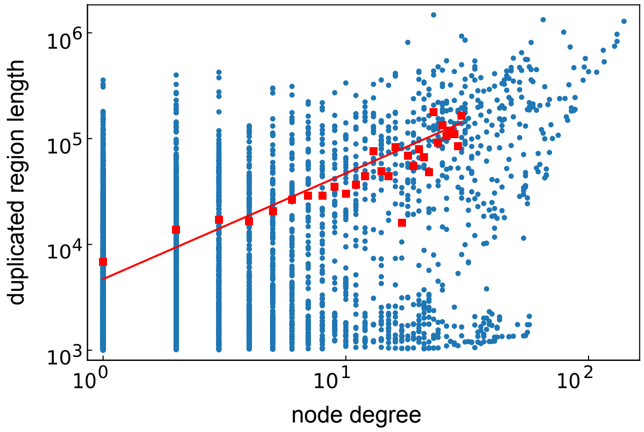

Sup. Figure 5 The length (in bps) for all duplicated regions (nodes of the SD network) plotted against the node degree on a log-log scale (blue dots). Even though the observed dependence is complicated and not linear the average length of a duplicated region turns out to grow linearly with a node degree (red dots). The red line represents a linear growth on a log-log scale.

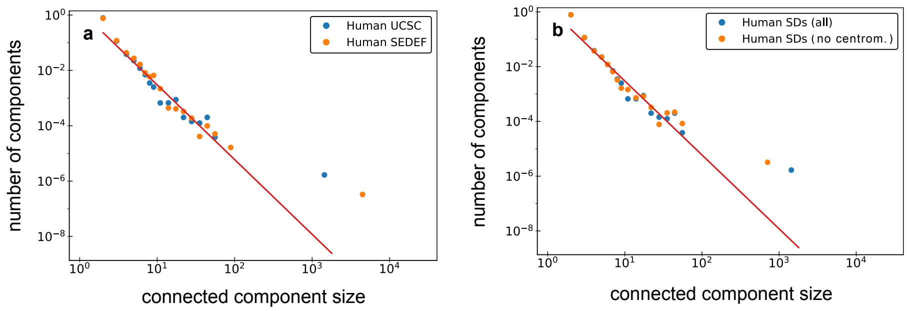

Sup. Figure 6 The connected component size distributions plotted on a log-log scale with logarithmic binning. **a** Comparison of the SD networks constructed from the UCSC annotated and SEDEF predicted SDs. **b** The SD networks constructed based on all SDs of the human genome and all SDs excluding pericentromeric ones (± 3 Mbs). In both cases the network topologies do not change substantially in characteristics we study.

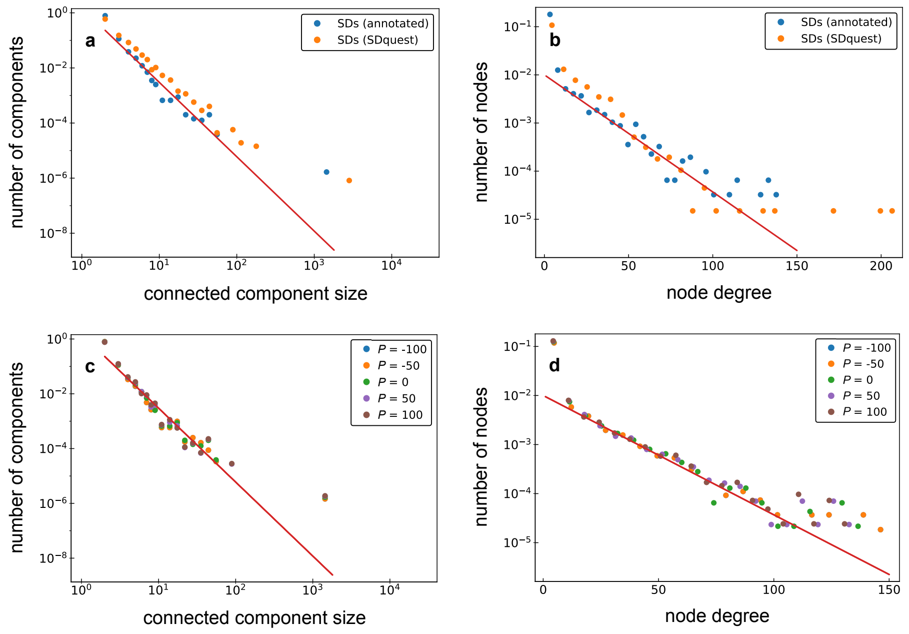

Sup. Figure 7 The figure represents comparison of different topological features of our network with ones observed in SD networks constructed in different manner. We can see the connected component size (**a**) and node degree (**b**) distributions of normal SD network and the one constructed based on duplications predicted with SDquest [5]. Those SDquest annotated SDs were filtered according to "softer" cut-offs (length > 500 bps, sequence identity $> 70\%$) which allow us to include older duplications into analysis. Even though the size of alternative SD network is substantially larger we can see that distributions are similar both in terms of slopes and giant component presence. The connected component size (**c**) and node degree (**d**) distributions of multiple SD networks with paddings P are plotted. This parameter represents number of bases added to extend (if $P > 0$) or shorten (if $P < 0$) each duplication interval on both sides before constructing an alternative SD network. The value of $P = 0$ corresponds to our normal SD network. We can see that even with quite large values of P parameter both distributions stay pretty much unchanged in all networks. The connected component size and node degree distributions are plotted on log-log and log-linear scales respectively.

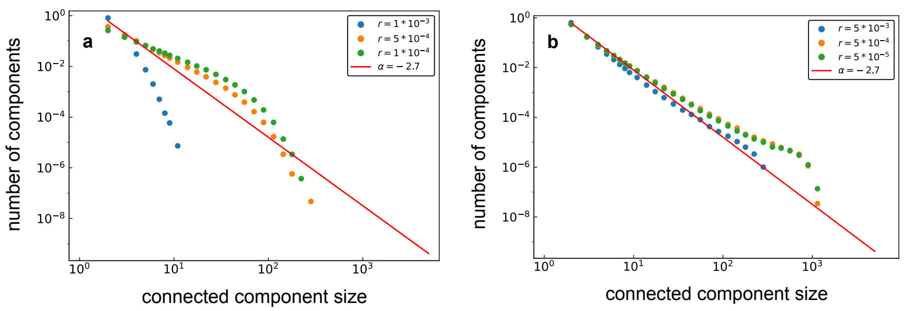

Sup. Figure 8 The connected component size distributions observed in simulations of network growth are plotted on a log-log scale. Additional process of edges loss was added in both UCM (**a**) and PCM (**b**) to check how it affects the topology of resulting synthetic networks. At each time step each edge is removed with the probability r (values of r are listed in the legends of the figures). Red line represents the slope of the distribution observed in the SD network. In one simulation (UCM with $r = 1 * 10^{-3}$) we can see a network that can not grow under such a large probability of edges loss r. One can see that when using reasonable values of r (too large values of r can hinder any network growth) both models of network growth behave as expected in standard UCM and PCM simulations.

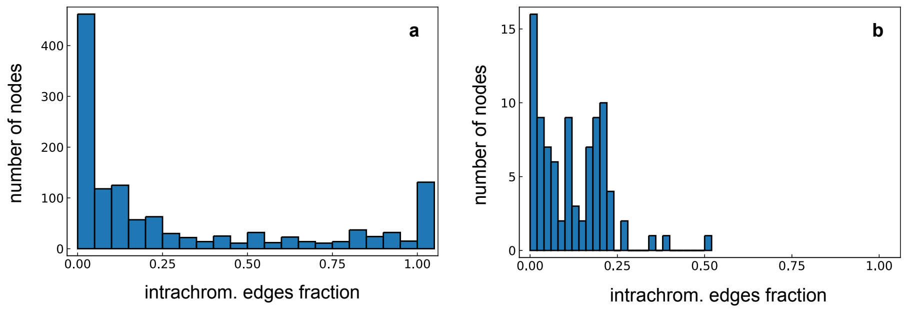

Sup. Figure 9 The histogram illustrates the distribution of fractions of intrachromosomal edges from all edges of nodes. Nodes that are prone to intrachromosomal duplications have values close to 1, while those with predominantly interchromosomal duplications are closer to 0. In (**a**) we include nodes with > 5 neighbors, while in (**b**) actively duplicating nodes with > 50 neighbors are included. We can see that the first distribution is bi-modal with two peaks around 0 and 1. Nodes with high node degree from the second histogram are, on the other hand, depleted with intrachromosomal hotspots.

Supplementary tables

SD networks:	Nodes	Edges	Intra- (%)	Tandem (%)	Shortest path	Clustering
original SDs ($P = 0$)	6656	16042	29	9	4.93	0.57
SDquest SDs	9605	34986	22	4	5.62	0.17
padding ($P = -100$)	7281	17166	30	10	4.89	0.58
padding ($P = -50$)	7266	17155	30	10	4.89	0.58
padding ($P = 50$)	6322	15550	27	8	4.83	0.56
padding ($P = 100$)	6213	15423	29	8	4.87	0.56

Sup. Table 1 Characteristics of several alternatively constructed SD networks are compared. The normal SD network that we used everywhere by default, several SD networks with different paddings P used in construction and the SD network built on SDquest predicted SDs (see Methods). The characteristics include: number of nodes and edges, fraction of intrachromosomal and tandem edges among all edges, a mean clustering coefficient and an average shortest path length. We can see that all characteristics of SD networks are stable when using different paddings P (the original SD network was constructed with $P = 0$). The SD network constructed on SDquest annotated SDs is larger (as expected with reduced cut-offs), however similar in other characteristics except for a mean clustering coefficient. It is way smaller than the one observed in the normal SD network (0.17 with 0.57). Curiously, this value is way closer to the one we observed in PCM simulations (Table 1). When we use SDs with length and sequence identity cut-offs - we artificially exclude "worse" (shorter and less conservative) alignments that are, on the other hand, present among SDquest predictions. This might artificially inflate the mean clustering coefficient observed in the normal SD network, while the values observed for the SDquest SD network and in PCM simulations are very close (0.17 with 0.18).

Supplementary text

In this section we present some analytical solution for key distributions of our models introduced in the main text.

1). In the UCM (Uniform Copying Model) each component grows with the rate proportional to its size (number of nodes). So a component size N as a function of an absolute time t and the time when a component was added to a network s changes in the following way:

$$\frac{\partial N(s,t)}{\partial t} = \delta N,$$

This differential equation can be solved by the following ansatz:

$$N(s,t) = 2e^{\delta(t-s)}, \qquad N(s=t,t) = 2$$

which can be inverted and solved for s:

$$s(N,t) = t - \frac{1}{\delta}\ln(\frac{N}{2})$$

This finally gives us the power-law distribution $p(N) \propto N^{\alpha}$ where $\alpha = -1$:

$$p(N,t) \propto \frac{\partial s(N,t)}{\partial N} \quad \propto \quad N^{-1}$$

2). In the PCM (Preferential Copying Model), on the other hand, each component $C(N,E)$ grows with the rate proportional to the sum of node degrees of all N nodes in a component which equals $2E$. According to our simulations $E \propto N^{1+f}$ dependence is characteristic for components in the PCM growth thus the size $N(s,t)$ of PCM components changes in the following way:

$$\frac{\partial N(s,t)}{\partial t} \propto \delta N^{1+f},$$

This differential equation can be solved in the following way:

$$N(s,t) \propto (C - \delta f(t-s))^{-1/f},$$

it can be inverted:

$$s - t \propto \frac{N^{-f} - C^{*}}{\delta f},$$

This leads to the power-law distribution $p(N) \propto N^{\alpha}$ where $\alpha = -1 - f$:

$$p(N,t) \propto \frac{\partial s(N,t)}{\partial N} \quad \propto \quad N^{-1-f}$$

C and C^{*} are constants.

3). When a node is duplicated in UCM component $C(N,E)$ an expected number of edges increases by $1 + f\,2E/N$, i.e. one edge to the daughter node plus an additional

fraction of f edges of the average node degree $2E/N$, since all nodes are duplicated equally likely. Therefore the number of edges E in UCM components changes with N in the following way:

$$\frac{dE(N)}{dN} = 1 + f\frac{2E}{N}$$

Firstly, the homogeneous differential equation is rearranged:

$$\frac{dE}{E} = 2f\frac{dN}{N},$$

and solved:

$$E(N) = CN^{2f}$$

Now we return to solving the original non-homogeneous equation for $C(N)$ using the variation of parameters method:

$$\frac{dC}{dN}N^{2f} + 2fCN^{2f-1} = 1 + 2fCN^{2f-1},$$

which can be reduced to:

$$dC = N^{-2f}dN,$$

and solved for $C(N)$:

$$C(N) = \begin{cases} \frac{1}{1-2f}N^{1-2f} + C^*, & f \neq 0.5 \\ \log N + C^*, & f = 0.5 \end{cases}$$

which leads to the following solution of the original differential equation:

$$E(N) = \begin{cases} \frac{1}{1-2f}N + N^{2f}C^*, & f \neq 0.5 \\ N\log N + NC^*, & f = 0.5 \end{cases}$$

Thus when $N \to \infty$ the number of edges E in the UCM components follows:

$$E(N) \propto \begin{cases} N, & 0 \leq f < 0.5 \\ N\log N, & f = 0.5 \\ N^{2f}, & 0.5 < f \leq 1 \end{cases}$$

C and C^* are constants.
